# Retraction Note: Influence of COVID-19 on the poultry production and environment

**DOI:** 10.1007/s11356-026-37787-9

**Published:** 2026-05-07

**Authors:** Hafez M. Hafez, Youssef A. Attia, Fulvia Bovera, Mohamed E. Abd El-Hack, Asmaa F. Khafaga, Maria Cristina de Oliveira

**Affiliations:** 1 Institute of Poultry Diseases, Faculty of Veterinary Medicine, Free University Berlin, Berlin, Germany; 2 Department of Agriculture, Faculty of Environmental Sciences, King Abdulaziz University, P.O. Box 80208, Jeddah, 21589 Saudi Arabia; 3 The Strategic Center to Kingdom Vision Realization, King Abdulaziz University, P.O. Box 80200, Jeddah, 21589 Saudi Arabia; 4 Animal and Poultry Production Department, Faculty of Agriculture, Damanhour University, Damanhour, 22516 Egypt; 5 Department of Veterinary Medicine and Animal Production, University of Napoli Federico II, via F. Delpino 1, 80137 Naples, Italy; 6 Poultry Department, Faculty of Agriculture, Zagazig University, Zagazig, 44511 Egypt; 7 Department of Pathology, Faculty of Veterinary Medicine, Alexandria University, Edfina, 22758 Egypt; 8 Faculty of Veterinary Medicine, Rio Verde University, Rio Verde, 75901-970 Brazil

**Retraction Note: Environmental Science and Pollution Research (2021) 28:44833-44844**

10.1007/s11356-021-15052-5

The Editor-in-Chief and the Publisher have retracted this article as the editorial and peer review processes appear to have been compromised. Based on this, the Editor-in-Chief no longer has confidence in the results and conclusions of the article.

Authors Youssef A Attia, Fulvia Bovera, Mohamed E. Abd El-Hack, Asmaa F. Khafaga and Maria Cristina de Oliveira disagree with this retraction. Author Hafez M Hafez did not respond to correspondence from the Publisher about this retraction.

